# Compliance of young children with ITN protection in rural Burkina Faso

**DOI:** 10.1186/1475-2875-5-70

**Published:** 2006-08-14

**Authors:** Claudia Frey, Corneille Traoré, Manuela De Allegri, Bocar Kouyaté, Olaf Müller

**Affiliations:** 1 Department of Tropical Hygiene and Public Health, Ruprecht-Karls-University Heidelberg, INF 324, 69124 Heidelberg, Germany; 2 Ministry of Health, Ouagadougou, Burkina Faso; 3 Centre de Recherche en Santé de Nouna (CRSN), BP 02, Nouna (Kossi), Burkina Faso

## Abstract

**Background:**

Insecticide-treated bed nets (ITNs) are known to be highly effective in reducing malaria morbidity and mortality. The effectiveness of ITNs is largely influenced by behavioural factors and not much is known regarding such factors under programme conditions.

**Methods:**

This descriptive study was nested into a large ITN effectiveness study in rural Burkina Faso. During two cross-sectional surveys in the dry and rainy season of 2003, random samples of young children from nine representative villages (n = 180 per survey) were investigated for compliance with ITN protection and related behaviour. Data were collected through direct observations and through interviews with mothers.

**Results:**

ITNs were perceived as very important for protection against mosquitoes and malaria particularly during the rainy season, but there were problems with their use during the dry season. Young children usually slept with their mother under the ITN and self-reported compliance was 66% and 98% during dry and rainy season, respectively (confirmed by direct observation in 34% and 79%, respectively). Important reasons for low compliance during the dry season were high temperatures inside houses and problems related to changing sleeping places during the night.

**Conclusion:**

Under programme conditions, compliance with ITN protection in young children is sufficient during the rainy season, but is rather low during the hot and dry season. Greater emphasis needs to be placed on information/education efforts to make people aware of the fact that the risk of contracting malaria may persist throughout the year.

## Background

Malaria has accompanied humankind for as long as 5,000 years, the first written evidence of the disease dating as far back as 2,700 BC in the Chinese medical classic *Nei Chin *(the Canon of Medicine) [1]. Protection techniques against insects have also been known for ages and were used long before the malaria transmission process was discovered [2]. Among the oldest approaches to prevent mosquitoes from biting was the use of bed nets and curtains which in the Roman and Greek ancient world simply meant the spreading of gauze and muslin curtains over places to protect. Additionally, the ancient Persians were said to destroy insects by using a powdered dried flower of a Dalmatian pyrethrum [3].

In recent years, these two techniques have been combined into the powerful tool of insecticide-impregnated bed nets and curtains (ITNs). ITNs have consistently been shown to be very effective and sustainable in reducing malaria morbidity and all-cause mortality in children of different malaria endemic areas [4-7]. They have also been shown to be highly cost-effective and are actually one of the most affordable control tools [8,9]. Moreover, the successful development of long-lasting ITNs avoids the regular re-treatment of ITNs, every 6 to12 months, which was accompanied by a notoriously low compliance [10,11]. ITNs are considered one of the most important intervention of the global "Roll Back Malaria" partnership [12].

The impact of ITN interventions under real life conditions is known to be influenced by a number of important socio-economic, cultural and ecological determinants [13-16]. Most published reports are based on KAP (knowledge, attitudes, practice) surveys. Thus, research which is conducted prior to the intervention rarely addresses crucial questions such as compliance during the programme's course [17-22]. This paper presents findings from two cross-sectional surveys (dry and rainy season) on the compliance of young children with ITN protection, conducted during a major ITN effectiveness trial in rural Burkina Faso.

## Methods

### Study area

The study took place in the research area of the *Centre de Recherche en Santé de Nouna (CRSN)*, Burkina Faso between May and August 2003 [7]. Nouna, the capital of the Kossi Province, is situated about 280 km northwest of Ouagadougou. At the time of the study, the CRSN study area included Nouna town (a population of approximately 25,000) and a rural area of 41 villages (a population of approximately 35,000) [23]. The area itself is a dry orchard savanna, populated by subsistence farmers of different ethnic groups.

The rainy season usually lasts about five months from June to October with 600 mm to 800 mm of total rainfall. During this period the whole area becomes flooded, with the development of suitable *Anopheles *breeding sites, favouring the *Anopheles gambiae *complex and causing entomological inoculation rates (EIR) of 100 to 1,000 infected bites per person per year [24]. The dry season comprises both a cold dry season (November until February) and a hot dry season (March until May). The area is holoendemic for malaria, with most of the transmission taking place during or briefly after the rainy season [25].

### Study design

This descriptive study was nested into a large ITN effectiveness trial which included all children of the rural CRSN study area born between 2000 and 2003 [7]. A total of 3,400 newborns from 41 villages were enrolled into this study and followed up for the long-term efficacy of ITN protection until their 5^th ^birthday. Children were individually randomized to ITN protection from birth until their 5^th ^birthday (group A) versus from month six until their 5^th ^birthday (group B). A prototype long-lasting ITN (PermaNet^®^) was used which was shown to be not really long-lasting [26]. As a consequence, all study ITNs were regularly re-treated with deltamethrin before the following rainy season [7]. Morbidity was followed up in cohort children of six sentinel villages (n = 420), while mortality was followed up in all study villages using a mix of study-specific surveillance and the existing demographic surveillance system (DSS) [23,7]. Field staff was permanently based only in the six sentinel villages, but specific village informants had been selected to facilitate the collaboration between the population and the ITN trial study team in all 41 study villages.

This study comprised two cross-sectional surveys in nine representative villages of the overall 41 villages included in the ITN trial. Surveys took place in May 2003 (hot dry season) and July/August 2003 (rainy season). Results are based on answers to a comprehensive questionnaire combined with unannounced visits during sleeping hours.

### Study participants

After stratification for the three health-centre defined sub-areas, the nine study villages were selected from the 41 trial villages (excluding the six sentinel villages) to represent the study population in its socio-cultural, demographic and geographical diversity. In each of the nine survey villages and independently for the dry and rainy season survey respectively, 20 ITN trial children were randomly selected for this study from the list of the trial participants (n = 180 per survey).

### Study procedures

The surveys in the nine villages always started by direct observations during an unannounced visit of a trained field worker to the respective study household, who was accompanied by the responsible village informant. These visits took place in the late evening (around 9.00 PM) or early morning (around 5.00 AM), when young children are expected to sleep. During visits, the utilisation of ITNs was verified and noted down on a standardized form and additionally documented with a digital camera. At the same time, the families were informed about the timing of the research team's visit on the following day, when the forms and the digital camera were then handed over to the research team upon their arrival.

A pre-tested structured questionnaire was then administered by the research team, consisting of a trained fieldworker and the investigator (CF), to the mothers/caretakers of the study children. In addition to demographic and socio-economic parameters, the questions addressed mainly aspects of behaviour regarding malaria prevention and treatment with particular emphasis on specific ITN use.

ITN compliance was evaluated by the question '*Did the study child sleep under the ITN last night*?' as well as by the direct observations during the respective nights.

### Data management

Data entry was done by the investigator (CF) directly after the survey visits. The data were analysed with EpiInfo 3.0 (October 2003) with simple proportions used to describe the parameters investigated.

### Ethical aspects

The study protocol was approved by the Ethical Committee of the Heidelberg University Medical School and the local Ethical Committee in Nouna, Burkina Faso. Informed community consent was sought through explanations and discussions during village meetings before the study was implemented. Oral informed consent from interviewees was a prerequisite for the interviews and the direct observations.

## Results

### Study population

Table 1 shows the demographic characteristics of the study population. Approximately half of the study children were male and belonged to group A, with no differences between surveys. The mean age of study children was 23.1 months (range: 2 – 37 months). There were no major differences between children surveyed during the dry and rainy season time points with regard to age and ethnicity.

**Table 1:** Characteristics of the study populations by season

	**Dry Season (*n *= 180)**	**Rainy Season (*n *= 180)**
**Intervention group**		
A	92 (51%)	82 (46%)
B	88 (49%)	98 (54%)
**Sex**		
Male	94 (52%)	93 (52%)
Female	86 (48%)	87 (48%)
**Ethnicity**		
Bwaba	28 (16%)	30 (16%)
Marka	52 (29%)	59 (33%)
Mossi	42 (23%)	37 (21%)
Peulh	53 (29%)	48 (26%)
Other	5 (3%)	6 (4%)
**Age**		
0 – 6 Months	3 (2%)	1 (1%)
7 – 12 Months	23 (13%)	30 (17%)
13 – 24 Months	58 (32%)	74 (41%)
25 – 36 Months	96 (53%)	69 (38%)
> 36 Months	0	6 (3%)

### Methods for mosquito and malaria control

The study ITNs were found in all of the 360 households visited during the two surveys. Beside the one ITN given per household for protection of the cohort child against mosquitoes, 85/180 (47%) and 75/180 (42%) of respondents reported the existence of at least one additional, but untreated bed net in their household during the dry and the rainy season time points respectively. Respondents indicated that other physical barriers commonly employed to prevent mosquitoes from biting were covering clothes and/or light blankets, which were mentioned by virtually all interviewees. Moreover, 158/180 (88%) and 171/180 (95%) reported the use of insecticide coils or insecticide spray in their households during the dry and the rainy season time points respectively. Traditional methods such as using specific plants as mosquito repellents were mentioned by some 10% of caretakers, both during dry and rainy season time points. 4/180 (2%) and 23/180 (13%) of respondents mentioned evacuation of stagnant water for mosquito control during the dry and the rainy season time points respectively.

The use of modern and traditional malaria prevention medications was reported by ten percent of respondents. Chloroquine and antipyretics were the modern drugs used at irregular intervals for prevention, while specific plants for drinking and bathing were mentioned as traditional prevention methods.

### Perceived advantages and disadvantages of ITN

The perceived advantages and disadvantages of ITN and bed net use in general were to a large degree dependent on the season. Table 2 shows the perceived advantages by season. Overall, a considerable proportion of respondents highly valued ITN with regard to protection against mosquitoes, other insects, larger animals (e.g. rats, snakes), malaria and other diseases, cold and dust, but also with regard to economic aspects of disease and insect control (reduced expenses due to less need for malaria drugs and insecticides). Most of these effects were more pronounced during the rainy season.

**Table 2:** Perceived advantages of ITN use in survey villages by season

**Advantage**	**Dry Season (*n *= 180)**	**Rainy Season (*n *= 180)**
Protection against mosquito nuisance	180 (100%)	180 (100%)
Protection against malaria	135 (75%)	150 (83%)
Protection against other diseases	151 (84%)	177 (98%)
Protection against wind/cold	141 (78%)	153 (85%)
Protection against dust/trash	58 (32%)	96 (53%)
Protection against insects	116 (64%)	179 (99%)
Protection against larger animals	43 (24%)	76 (42%)
Intimacy	14 (8%)	13 (7%)
Economy of malaria drugs/insecticides	5 (3%)	73 (41%)

While 105/180 (58%) of respondents complained about the disadvantage of heat and the corresponding lack of convenience during the dry season, this proportion was only 15/180 (8%) during the rainy season. Practicability was influenced by the tendency to sleep outside the house during the dry season, the problems in fixing and keeping the bed net outside and the difficulty to remount the net in the early morning hours when many people withdraw inside the house due to falling temperatures. As a consequence, mothers and young children often slept either unprotected during the first or the second half of the night depending on where the bed net was mounted initially, the majority of them preferring to leave the net mounted in the house and to retire underneath when moving indoors early in the morning. Other stated reasons for not using the ITN during the dry season were the lack of mosquito nuisance in 13/180 (7%) and travelling in 5/180 (3%) of respondents.

### ITN compliance

Table 3 shows the directly observed and the caregiver-reported compliance with ITN in young children by season. Observed as well as reported ITN use was much higher in the rainy season compared to the dry season. ITN use was always considerably over-reported compared to the observed ITN use, but this difference was more marked in the dry season. Part-time use of the ITN was only declared during the dry season survey.

**Table 3:** Compliance with ITN use in study children by season

	**Dry Season (*n *= 180)**	**Rainy Season (*n *= 180)**
Self-reported full-time ITN use during last night	118/180 (66%)	177/180 (98%)
Self-reported part-time ITN use during last night	21/180 (12%)	0 (0%)
Observed ITN use during last night	62/180 (34%)	142/180 (79%)

Of the dry season respondents who clearly reported that the study child was not under ITN protection during the previous night, approximately half stated that the ITN had not been used for less then one week while one quarter gave periods up to one month and the other quarter for more than one month. The three rainy season respondents, who had reported that the child was not protected with the ITN during the previous night, all declared that this was an exception and that their children were usually sleeping under the ITN.

The reasons given for only part-time protection of children during the previous night of the dry season were mainly prolonged household activities during which the child is commonly carried on the back of the mother. Additionally, due to high temperatures inside the house where the ITN is situated, mothers and their children tend to sleep outside during the first night hours but few mount their net there. In those cases where the ITN was successfully installed outside the house, children were unprotected in the cold morning hours when the mothers moved back with them inside the house where nets were generally not remounted.

Direct observation revealed that people were very creative in fixing the ITNs both inside and outside the house. The great majority of the villagers usually managed to mount their nets correctly, only a few reported to sometimes use the ITN for a limited time period as a sort of "cover cloth" for the child when they do not have sufficient time to install it immediately. Of those who reported full compliance with ITN use, 67/118 (57%) and 117/177 (66%) said to tack it correctly under the mat or mattress during dry and rainy season surveys respectively. The proportion of ITN found to be tacked correctly under the mat/mattress during direct observations was 6/62 (10%) and 48/142 (34%) during the dry and rainy season surveys respectively.

### ITN sleeping patterns

During the dry season, 104/180 (58%) of the study children slept primarily on mats set outside the house. Only a few households reported moving the actual beds outdoors. Children, however, were reported to use both mats and beds, as they were often moved between indoors and outdoors in the course of the night. This pattern was also confirmed by the direct observations. During the rainy season instead, 135/180 (75%) of children consistently slept on beds inside the house.

None of the study children slept alone under the ITN, instead they shared it in most occasions with their mother and eventually other siblings and only exceptionally with their father (Table 4). When children were found alone under the ITN during unannounced visits this was explained by their caretakers being busy with household activities. There was no evidence that an ITN had been taken away from the study child to be used solely by other persons in the household.

**Table 4:** Sleeping pattern of study children by season

	Dry Season (*n *= 118)*	Rainy Sason (*n *= 177)*
Child + mother	59 (50%)	78 (44%)
Child + mother + 1 sibling	52 (44%)	80 (45%)
Child + mother + 2 siblings	4 (3%)	12 (7%)
Child + mother + father	2 (2%)	6 (3%)
Child + father	1 (1%)	0

*only children reported to be compliant with the ITN

During the dry season, 99/118 (84%) of children were reportedly put under the ITN after dinner (usually between 7.00 and 8.00 PM) and 106/118 (90%) did stay there until sunrise (usually between 6.00 and 7.00 AM). During the rainy season, the corresponding figures were 150/177 (85%) and 161/177 (91%).

## Discussion

This study has explored factors related to the acceptance and use of ITN in a rural area of Burkina Faso and identified behavioural aspects that could influence the implementation of ITN in malaria control programmes. Unlike previous studies exploring compliance with ITN use [14,21,27-29], this study relied on the combined collection of self-reported information and direct observation. This method, which had found limited application only in early bed net trials [30-32], allows a better assessment of the extent to which self-reported information corresponds to people's actual behaviour. Therefore, the study provides further evidence that compliance with bed net use tends to be over-reported [30-32], especially during the dry season. One must consider, however, that in the study, compliance with bed net use was observed only at one specific point in time, making it impossible to exclude that the observation occurred just before the child was placed under the net or just after he/she had been removed from under the net.

The most encouraging finding emerging from the study relates to the fact that two years after the beginning of the trial, the ITNs which had been distributed were without exception still present in the 360 households visited on the occasion of this study. In addition, there was no evidence that an ITN had been taken away from the child to whom it had been assigned to be solely used by another household member. In virtually all instances, respondents reported that the ITN was jointly used by the study child, his/her siblings, and their mother. Such findings appear most favourable when considering that under similar circumstances, 28% of bed nets distributed free of charge in Burundi were found to be resold to neighbouring Tanzania [33].

In addition to the ITNs distributed as part of the trial, non-treated conventional bed nets were also found to be used by about 45% of study respondents. This figure confirms the net prevalence assessed at baseline prior to implementation of the ITN trial [34] and indicates that the study setting represents an African average. Net prevalence in fact has been found to vary across endemic regions from below 10% in Malawi, Zimbabwe, and Ghana [20,35,36] to over 90% in The Gambia and Mali [29,37].

The results from this study suggest that bed nets are used throughout the year, although important differences in compliance exist between the rainy and the dry season. Seasonal changes in temperature influence sleeping patterns and thus, as a consequence, people's bed net use. ITN compliance was found to be very high in the rainy season but to drop substantially in the hot dry season. Similar seasonal variations in the compliance with ITN use had already been observed across different endemic regions, indicating that bed net use may vary between 20 and 76 per cent during the hot dry season to over 90 per cent during the rainy season [14,21,27-29].

Asking respondents to motivate their decision to sleep or not to sleep under the net allowed a better understanding of possible obstacles to the continuous compliance with ITN use. Since children sleep with their mothers, changes in adult sleeping preferences and patterns across seasons inevitably influence whether the child is protected throughout the night. This study indicates a certain easiness to comply with ITN use during the rainy season when people sleep indoors. It also confirms previous findings citing heat is an obstacle to sleep indoor and under a net [19,38-40] and a preference to sleep outdoors during the hot dry season [28]. In addition, however, our study also deepens existing knowledge by detailing that even during the hot dry season, people may not sleep exclusively outdoors, but may move in and out of their house, depending on temperature changes during the course of the night. A similar sleeping pattern with varying sleeping places throughout the night had been found in northeast Ghana, but, though being discussed as a potential problem, was not considered to hamper bed net use [36]. Thus, the phenomenon of changes in sleeping places seems to be more widespread than supposed earlier, making it difficult for users to mount and dismount the net unless manufacturing techniques are modified to make the mounting and dismounting of the nets simpler and more convenient for users [41].

The totality of respondents in the study reported protection against mosquito nuisance as the primary reason for sleeping under a net. In line with findings from prior research, such wish to protect oneself from mosquito nuisance is also likely to explain why in combination with ITN use, people reported using a variety of other means of protection against mosquitoes, ranging from covering themselves with thick clothes and blankets [42,43] to using both traditional and modern insecticides [20,29,34,39]. Other reasons mentioned for using ITNs were protection against diseases, in particular malaria; protection against insects and small animals in general; protection against dust and the cold; and a wish to create an intimate setting. In line with previous research, these findings confirm that ITN use is motivated by the co-existence of a variety of reasons [33,39,44-46].

An interesting feature emerging from the study is the high percentage of respondents reporting that ITNs offer protection against malaria. In addition, a large percentage of respondents reported having spent less money on insecticides and malaria drugs following ITN use. This high awareness that ITN use reduces malaria transmission is most likely due to the fact that the distribution of the ITNs took place in an area which, thanks to a series of malaria studies including the ITN effectiveness trial, has been sensitized over a number of years on the importance of using ITNs to protect children from malaria. This finding represents an encouraging element for the development of communication and sensitization campaigns as it shows that when repeatedly and correctly informed, people do appreciate novel information on the advantages of ITN use even when malaria transmission may not necessarily be conceptualized in relation to mosquito bites [34,47,48].

Such high awareness that ITNs offer protection against malaria could potentially be used to enhance compliance with ITN use also during the hot dry season. Compliance with ITN use is in fact likely to drop in the hot dry season as net use becomes rather inconvenient due to climatic changes and as the incentive to protect oneself from mosquito nuisance is reduced given the lower mosquito density [14,29,49]. At such moment in the year, however, people could be motivated to use ITN if they gained appreciation of the fact that although not as high as during the rainy season, the risk of contracting malaria persists throughout the year. The fact that respondents reported the use of both traditional and modern remedies at intermittent intervals as malaria prophylaxis offers an additional powerful indication of how people appreciate the need to prevent the disease and are willing to invest to adopt behaviours which are conducive towards such prevention.

## Conclusion

The effectiveness of ITN interventions depends to a large degree on human behaviour as a factor in health, influenced by socio-cultural traditions as well as economic and environmental determinants. The failure of previous malaria control programmes has been attributed to the lack of adequate consideration given to these aspects which must be taken into account and should be routinely incorporated as an essential feature into control programmes.

In conclusion, compliance with ITN protection in young children under programme conditions was shown to be sufficient during the rainy season but rather low during the hot and dry season in a malaria holoendemic area of Burkina Faso. Greater emphasis needs to be placed on information/education efforts to make people aware of the fact that the risk of contracting malaria often persists throughout the year.

## Authors' contributions

C Frey, C Traoré, B Kouyaté and O Müller designed the study. C Frey and C Traoré conducted and supervised the field work. C Frey, M De Allegri and O Müller analysed the data. All authors contributed to the writing of the paper. O Müller was the principal investigator.
